# Activity of Eribulin in a Primary Culture of Well-Differentiated/Dedifferentiated Adipocytic Sarcoma

**DOI:** 10.3390/molecules21121662

**Published:** 2016-12-03

**Authors:** Alessandro De Vita, Giacomo Miserocchi, Federica Recine, Laura Mercatali, Federica Pieri, Laura Medri, Alberto Bongiovanni, Davide Cavaliere, Chiara Liverani, Chiara Spadazzi, Dino Amadori, Toni Ibrahim

**Affiliations:** 1 Osteoncology and Rare Tumors Center, Istituto Scientifico Romagnolo per lo Studio e la Cura dei Tumori (IRST) IRCCS, Via Piero Maroncelli 40, 47014 Meldola (FC), Italy; giacomo.miserocchi@irst.emr.it (G.M.); federica.recine@irst.emr.it (F.R.); laura.mercatali@irst.emr.it (L.M.); alberto.bongiovanni@irst.emr.it (A.B.); chiara.liverani@irst.emr.it (C.L.); chiara.spadazzi@irst.emr.it (C.S.); dino.amadori@irst.emr.it (D.A.); toni.ibrahim@irst.emr.it (T.I.); 2 Pathology Unit, Morgagni-Pierantoni Hospital, Via Carlo Forlanini 34, 47121 Forlì, Italy; federica.pieri@auslromagna.it (F.P.); laura.medri@auslromagna.it (L.M.); 3 Unit of Surgery and Advanced Oncologic Therapies, Morgagni-Pierantoni Hospital, Via Carlo Forlanini 34, 47121 Forlì, Italy; cavalied@gmail.com

**Keywords:** eribulin, microtubule inhibitors, adipocytic sarcoma, primary culture

## Abstract

Eribulin mesylate is a novel, non-taxane, synthetic microtubule inhibitor showing antitumor activity in a wide range of tumors including soft tissue sarcomas (STS). Eribulin has been recently approved for the treatment of metastatic liposarcoma (LPS) patients previously treated with anthracyclines. This work investigated the mechanism of action of this innovative antitubulin agent in well-differentiated/dedifferentiated LPS (ALT/DDLPS) which represents one of the most common adipocytic sarcoma histotypes. A primary culture of ALT/DDLPS from a 54-year-old patient was established. The anticancer activity of eribulin on the patient-derived primary culture was assessed by MTT and tunel assays. Eribulin efficacy was compared to other drugs approved for the treatment of STS. Cell migration and morphology were examined after exposure to eribulin to better understand the drug mechanism of action. Finally, Western blot analysis of apoptosis and migration proteins was performed. The results showed that eribulin exerts its antiproliferative effect by the arrest of cell motility and induction of apoptosis. Our results highlighted the activity of eribulin in the treatment of ALT/DDLPS patients.

## 1. Introduction

Eribulin mesylate is a synthetic macrocyclic ketone analogue of the natural compound helichondrin B with an innovative microtubule-inhibitory action.

Eribulin was approved in 2010 by the United States Food and Drug Administration (FDA) as a monotherapy for patients with advanced/metastatic breast cancer pretreated with either an anthracycline- or taxane-based regimen [1]. The European Medicines Agency (EMA) Committee for Medicinal Products for Human Use (CHMP) has recently approved eribulin for the treatment of adult patients with unresectable liposarcoma (LPS) pretreated with an anthracycline-based therapy for metastatic disease.

This microtubule-depolymerizing drug exerts an antitumor activity through a mechanism of action different from any other known microtubule inhibitors [2].

Although several studies have demonstrated eribulin’s antitumor effects, its mechanism of action remains unclear. Preclinical and clinical studies have shown that eribulin seems to act via a mechanistically unique inhibition of microtubule dynamics, involving the site-specific binding of tubulin. This results in the suppression of microtubule polymerization and tubulin sequestration into non-functional aggregates, and subsequent cell cycle arrest. Other complex effects, including vascular remodeling, reversion of the epithelial-mesenchymal transition and suppression of migration and invasion have been recently described [3,4].

Soft tissue sarcomas (STS) are a heterogeneous group of rare tumors arising from soft tissue or bone with uncertain etiology and difficult classification. They represent around 1% of all adult cancers. To date, more than 50 histological subtypes of STS have been identified [5]. 

Adipocyte sarcoma or LPS is one of the most common STS subtypes, accounting for 15% of all sarcomas, with an incidence of 24% of all extremity and 45% of all retroperitoneal STSs [6]. The World Health Organization (WHO) classification system has divided LPS into four different subtypes: atypical lipomatous tumor (ALT), dedifferentiated liposarcoma (DDLPS), myxoid LPS (MLPS) and pleomorphic LPS (PLS) [7].

ALT and DDLPS are the most frequent subtypes, accounting for 40%–45% of all LSPs [8]. The amplification of the 12q13-15 chromosome region is one of the most important genetic events of these LPS subtypes. Several potential oncogenes including MDM2, CDK4, GLI, DDIT3, and HMGIC are located in this region. The importance of these features is confirmed by the FISH analysis of MDM2 gene amplification, which is currently performed for the standard differential diagnosis of ALT/DDLPS [9]. Surgical resection with negative margins represents the cornerstone for localized primary tumor, while chemotherapy and radiotherapy treatments in adjuvant and neo-adjuvant settings have shown controversial results. For unresectable disease the standard of care is represented by chemotherapy with very poor results [10,11].

In a phase II study performed by European Organisation for Research and Treatment of Cancer (EORTC), eribulin activity was assessed in patients with STS including several histotypes with no data available on the adipocytic sarcoma subgroups [12]. The following phase III trial with patients affected only by adipocytic sarcoma and leyomiosarcoma evaluated the role of eribulin against dacarbazine [13]. Results showed that the benefit of eribulin on overall survival (OS) was higher in adipocytic sarcoma than in leyomiosarcoma patients.

However, little data on eribulin activity for each LPS subtype has as yet been reported.

In this scenario, patient-derived specimens represent a valuable source for investigating the complex effect of the pharmacodynamics interaction of this antitubulin agent, especially in rare malignancies for which preclinical and clinical data are not complete.

We report here the activity of eribulin in a primary culture of ALT/DDLPS, one of the most common adipocytic sarcoma subtypes. This work investigated the mechanism of action of this innovative tubulin inhibitor to better understand its antitumor effect which represents the starting point for further investigations. To the best of our knowledge this is the first study to have assessed eribulin’s activity on ALT and DDLPS primary cultures.

## 2. Results

### 2.1. Patient History

A 54-year-old patient presented abdominal pain. Ultrasound imaging revealed an abdominal mass. A subsequent computed tomography (CT) scan confirmed the presence of an abdominal lesion of about 5.2 cm with a predominant adipose component located in the small intestine. A laparoscopic wedge resection of the lesion was performed, and the pathologic report revealed peritoneal ALT, with surgical negative margins. After surgery there was no evidence of metastatic disease and the patient started follow-up.

Five years later a control CT scan showed a nodular solid lesion of 3.9 cm in the previous surgical site. The patient underwent laparotomic resection of the lesion; the pathologic evaluation revealed DDLPS with low-grade spindle cells (Fédération nationale des centres de lutte contre le cancer (FNCLCC) grade 3) with focally positive surgical margins. MDM2 gene amplification was detected by FISH analysis. After surgery, the patient underwent chemotherapy treatment with epirubicin plus ifosfamide for six cycles in another institute, after which follow-up was resumed.

One year later an additional CT scan showed a new abdominal recurrence with the presence of multiple solid lesions in the abdomen. Two months later a surgical resection of the peritoneal nodules was performed. The pathologic report showed the presence of one nodule of 6 cm close to the angle of Treitz positive for DDLPS, two nodules of 6.5 cm and 3.5 cm, respectively, in the left mesocolon positive for ALT lipoma like, and two additional nodules in the mesentery of 4.0 cm and 2.5 cm, respectively, positive for ALT lipoma like. MDM2 gene amplification was detected by FISH analysis.

### 2.2. Establishment of Patient-Derived Culture of Adipocytic Sarcoma

**Figure 1:**
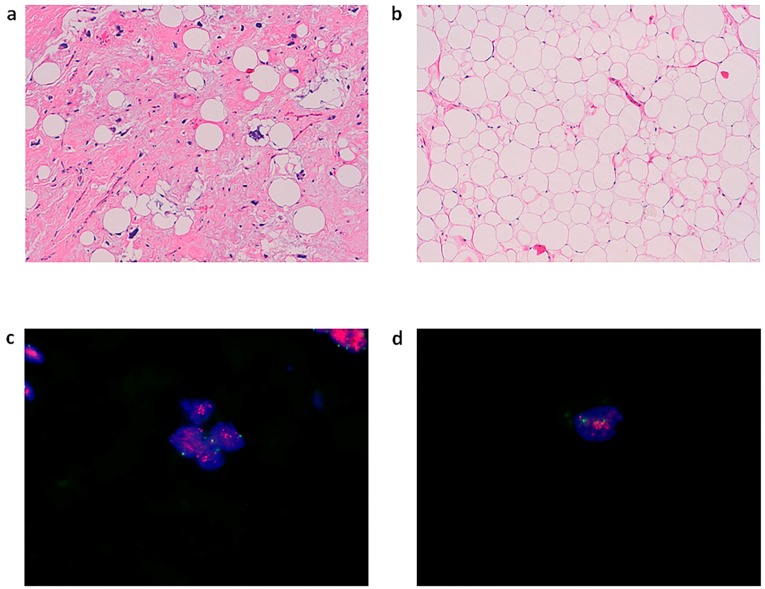
(**a**) HE 10× staining of the surgical specimen showing DDLPS (light-blue stroma) infiltrating adipose tissue rich in lipoblasts; (**b**) HE 10× staining of the surgical specimen showing DDLPS (light-blue stroma) infiltrating adipose tissue rich in lipoblasts; (**c**) MDM-2 amplification analysis of patient specimen performed by in situ hybridization at 100× magnification (Vysis MDM2/CEP12 dual color FISH probe kit); (**d**) MDM-2 amplification analysis of established primary culture performed by in situ hybridization at 100× magnification (Vysis MDM2/CEP12 dual color FISH probe kit).

Hematoxylin and eosin (HE) staining of the surgical material reviewed by an experienced sarcoma pathologist confirmed the diagnosis of ALT and DDLPS. DDLPS was characterized by adipose tissue rich in adipocytes with regions infiltrated by markedly atypical tumor cells in a fibrous stroma (Figure 1a). ALT lipoma like was characterized by mature lipoblasts uniformly arranged with no great variation in size (Figure 1b). MDM2 amplification assessed by FISH analysis was found positive, confirming the diagnosis (Figure 1c). The primary culture obtained from the surgical specimen continued to proliferate after several passages in monolayer cultures. Cytological analysis of MDM2 amplification confirmed the establishment of the patient-derived culture of ALT/DDLPS by 94.6% (158 out of 167 cells counted were found positive for MDM2 amplification; Figure 1d).

### 2.3. Eribulin Anticancer Activity on LPS Cells 

Eribulin’s effect on the viability of primary LPS cultures was assessed using the mitochondrial reduction assay MTT. The efficacy of eribulin treatment was compared to ifosfamide, epirubicin, and ifosfamide plus epirubicin, which represent the standard chemotherapy for LPS patients. 

**Figure 2:**
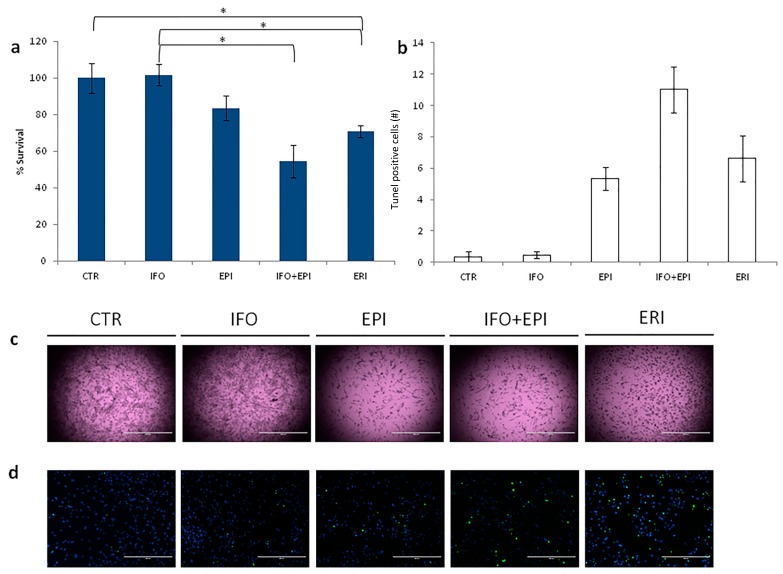
(**a**) Cytotoxicity assay of primary culture treated with: ifosfamide, epirubicin, ifosfamide plus epirubicin and eribulin. Differences between groups were assessed by a two-tailed Student’s *t*-test, and accepted as significant (*) at *p* < 0.05; (**b**) Number of tunel-positive cells (green spots) and live cells (blue spots); (**c**) 2× images of primary culture after the treatment, scale bar 2000 µm; (**d**) 10× images of tunel analysis showing apoptotic cells (green spots) and cell nuclei (blue spots), scale bar 400 µm.

**Figure 3:**
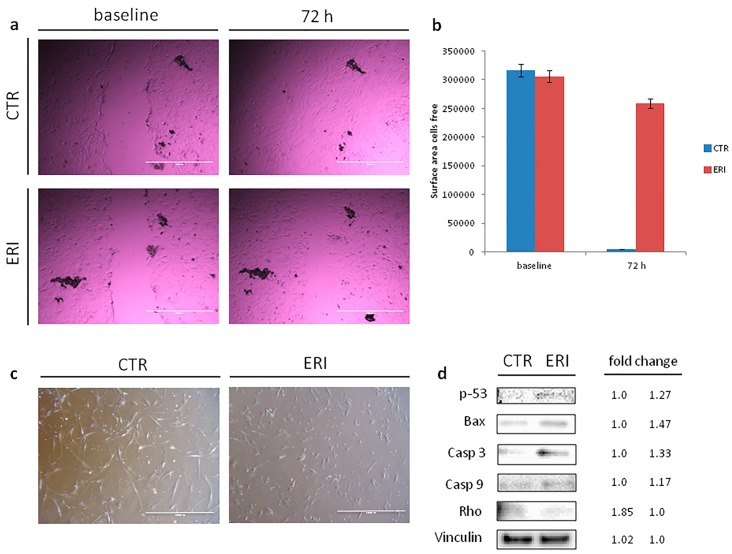
(**a**) Scratch wound assay. Wounds were generated after cell confluence. Cell migration and wound closure were assessed after 72 h, 4× images of primary culture (control sample and treated sample) at baseline and after 72 h, scale bar 1000 µm; (**b**) Areas of migration measured in the control sample and treated sample on days 0 and 3; (**c**) Morphological changes in the primary culture after eribulin treatment, 4× images, scale bar 1000 µm. After 72 h of treatment, eribulin induced a decrease in cell size, the cells lost some of the cytoplasmic architecture features, rounded up and were reduced in number; (**d**) Western blot analysis of apoptosis (p-53, Bax, Casp3 and Casp9) and migration-related proteins (Rho) and fold change in protein expression.

Patient-derived culture treated with eribulin showed 70% survival compared to untreated controls (Figure 2a). Ifosfamide had no effect on survival, while epirubicin and ifosfamide plus epirubicin reported 83% and 54% survival, respectively. Morphological changes in primary culture after eribulin exposure were observed under the optical microscope (Figure 3c). In particular, we observed that samples featured cell shrinkage, rounding-up, and a decrease in density. Apoptosis was evaluated by nick end-labeling (tunel) analysis to assess the anticancer activity of the tested schedules. The number of apoptotic cells confirmed the data obtained by the previous chemosensitivity assay (Figure 2b,d).

### 2.4. Eribulin Inhibition of Cell Migration and Induction of Apoptosis

Cell migration and morphology were examined after treatment with eribulin to gain insight into how eribulin induces its antiproliferative effect. Cells were cultured for 72 h in the presence of eribulin and a scratch wound assay was performed to evaluate the migration ability when compared to untreated cells. As shown in Figure 3a, no migration was detected after exposure to eribulin (almost the same cell-free surface area at baseline and at 72 h), while in the control group the wound completely disappeared after 72 h (no cell-free surface area; Figure 3b). This data indicated that eribulin exerts an antitumor activity through a mechanism entailing inhibition of cell motility.

### 2.5. Eribulin Modulation of the Expressions of Apoptosis and Migration-Related Proteins

To further determine how eribulin induces apoptosis and arrests migration, the expression levels of apoptosis and migration-related proteins were evaluated by Western blot analysis (Figure 3d). Figure 3d shows how eribulin upregulated the expression of p-53 protein after 72 h treatment. The effect of eribulin on Bax was also examined to investigate the mechanism involved in executing apoptosis. Bax expression was upregulated with a fold change of 1.46 compared to the control, confirming the drug-mediated induction of apoptosis. Since the levels of p-53 and Bax proteins were promoted by eribulin, their downstream proteins were further examined. Casp-3 and Casp-9 were upregulated, confirming that eribulin induces apoptosis through the caspase-dependent apoptotic pathway. Rho expression was also reduced (1.85-fold change), indicating the inhibition of cell motility. These results additionally confirmed that eribulin induces cell apoptosis and inhibition of cell motility.

## 3. Discussion

STSs consist of a heterogeneous group of different rare tumors. LPS is the most common type of STS, accounting for 15% of all sarcomas [6]. The treatment of choice in localized disease is surgical resection with negative margins, whereas the use of adjuvant and neoadjuvant chemotherapy is still debated. In the metastatic setting the standard of care is chemotherapy, although with limited results [10,11]. Novel compounds such as trabectedin and eribulin have been recently introduced with encouraging preliminary results. A better understanding of the mechanism of action of these drugs is crucial for transferring these new treatment options into clinical practice.

Primary culture represents a valuable tool extensively used in translational research of tumor pathophysiology, pharmacology and other related subjects [14,15,16,17]. The establishment of a primary culture of cells from surgically-resected tumor tissue represents a promising strategy for designing more suitable treatments, in particular for rare tumors with poor clinical outcomes such as STS [10]. 

This work evaluated the antitumor effect of the novel microtubule inhibitor eribulin on an ALT/DDLPS near-patient primary culture. 

We established an ALT/DDLPS primary culture model. In particular, cytological amplification analysis of ALT/DDLPS-related marker MDM2 of the cells isolated from the tumor specimen corroborated the presence of cancer cells (Figure 1d).

Standard MTT assay and tunel analysis were performed for evaluating the sensitivity to eribulin of the ALT/DDLPS primary culture. Results demonstrated that eribulin exhibited favorable viability inhibition of the near-patient primary culture, confirming its activity in the treatment of ALT/DDLPS. In particular, compared to other standard treatments for adipocytic sarcoma, the antiproliferative activity of eribulin resulted in lower survival rates (Figure 2a). This was especially evident when compared to the well-known anthracycline epirubicin regimen (70% cell survival rate with eribulin (ERI) vs. 83% with epirubicin (EPI) *p* = 0.09). The combination regimen of ifosfamide (IFO) plus epirubicin was the most effective treatment (70% ERI vs. 54% IFO + EPI *p* = 0.05).

In the clinical setting, anthracyclines are among the most active drugs for the treatment of STS with a 16%–27% response rate as a single agent [18,19].

Doxorubicin-based combination chemotherapy regimens produce an increase in response rates, although with no improvement in overall survival (OS) [20,21,22].

A phase III trial evaluated the dose intensification of doxorubicin with ifosfamide compared to doxorubicin alone in metastatic STS patients in first-line treatment. The combination achieved a significantly higher response rate than doxorubicin alone (26% vs. 14%), although with no significant difference in OS [23]. A monotherapy regimen with anthracyclines may be considered in clinical practice for its lower toxic effects, especially for disease control purposes. Interestingly, these clinical results are consistent with our trial, although the latter covered all types of STS.

Ifosfamide alone did not affect the survival rate (70% ERI vs. 100% IFO *p* = 0.03), but results from the combination regimen (ifosfamide plus epirubicin) seemed to suggest a synergistic effect when ifosfamide is co-administered with the anthracycline. These findings were confirmed by the number of apoptotic cells obtained by tunel analysis (Figure 2b,d). 

Our study provided the first indication of treatment efficacy for some of the drugs currently used or under clinical evaluation for this adipocytic sarcoma subtype.

It is widely known that most microtubule-targeted antitumor compounds induce abnormal microtubule arrangement, leading to cell shape changes and limited proliferation capacity [24]. Morphological changes in culture such as cell shrinkage and rounding-up were detected after eribulin exposure (Figure 3c). These features indicated the action of a mechanistically unique inhibition of microtubule dynamics mediated by this agent. 

We also observed that eribulin can significantly inhibit cell motility and induce apoptosis. These two non-exclusive molecular mechanisms were shown as the triggers of cell growth and proliferation inhibition. Cell migration inhibition mediated by eribulin was demonstrated by the wound scratch assay (Figure 3a). The reduced expression of migration-related protein Rho suggested the exertion of the eribulin antiproliferative effect through a mechanism entailing cell motility inhibition. To the best of our knowledge, the role of eribulin as a suppressor of Rho expression has not yet been documented in LPS, representing one of the new possible mechanisms of action mediated by eribulin. 

**Figure 4:**
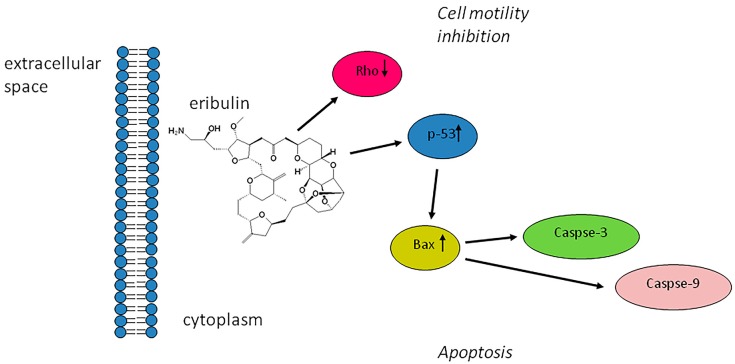
Possible mechanism of the cytotoxic effect mediated by eribulin in ALT and DDLPS patient-derived primary culture.

We demonstrated that the treatment of ALT/DDLPS resulted in the upregulation of proapototic proteins p-53 and Bax. This event leads to the collapse of the mitochondrial transmembrane potential and activates caspases which have a critical role in the mitochondrial-mediated apoptotic pathway. The examination of downstream protein Casp-3 and Casp-9 levels showed that eribulin could intensify their expression. All these experimental data proved eribulin’s ability to induce cell death program activation through the caspase-dependent mitochondrial pathway (Figure 4).

## 4. Materials and Methods

### 4.1. Establishment of Primary Cell Culture

A patient-derived adipocytic sarcoma cell culture was isolated from the surgical specimen.

Briefly, the tumor mass was surgically excised as reported in the result section. The protocol was approved by the Local Ethics Committee and performed in accordance with Good Clinical Practice and the Helsinki declaration. The patient gave his written informed consent to take part in the study.

The surgical specimen was analyzed by a pathologist and processed within 3 h of surgical resection.

The tumor specimen was washed twice in sterile phosphate buffered saline (PBS) and shredded into 1–2 mm^3^ pieces with surgical scalpels. The shredded pieces were incubated with a PBS solution of 2 mg/mL collagenase type I (Millipore Corporation, Billerica, MA, USA) at 37 °C in stirring condition for 15 min; the sample was then stored overnight at room temperature.

The day after collagenase digestion was blocked by the addition of DMEM supplemented with 10% fetal bovine serum, 1% glutamine and 10% Penicillin/Streptomycin.

The cells were isolated from the aggregates using 100 µm sterile filters (CellTrics, Partec, Münster, Germany). Cells were counted and seeded in standard monolayer cultures at 80,000 cells/cm^2^ density, and maintained in complete DMEM medium at 37 °C in a 5% CO_2_ atmosphere. 

### 4.2. Immunohistochemical Analysis

HE was performed for evaluating cell morphological features and distribution. Briefly, tissue specimen was recovered, washed twice with PBS, immediately embedded using paraffin in a cryomold (25 mm × 20 mm × 5 mm) and instantly frozen at −80 °C. Afterwards, 5-µm-thick slides were sectioned cutting paraffin-embedded tissue blocks with a microtome; the slides were hydrated and stained with ematoxylin (Sigma-Aldrich, Saint Louis, MO, USA) and eosin (Sigma-Aldrich). Finally, stained slides were washed with PBS 1× three times, mounted with Cytoseal™ XYL (Thermo Scientific Richard-Allan Scientific, Waltham, MA, USA) mounting media, covered with a coverslip, and analyzed.

MDM-2 amplification was performed for diagnosis purpose by FISH analysis according to the manufacturer’s instructions (Vysis MDM2/CEP12 dual color FISH probe kit). For primary culture 100,000 cells were cytospinned onto glass slides and MDM-2 amplification was detected by FISH analysis as previously described.

### 4.3. Protein Expression Analysis

Patient-derived cell cultures were exposed to eribulin at the concentration of the human plasma peak as specified in the drug testing section. After 72 h the cells were trypsinized and immediately frozen with liquid nitrogen and stored at −80 °C.

The proteins were then extracted using a RIPA buffer with 10% PMFS, 1% HALT phosphatase inhibitor cocktail and 0.5% Protease inhibitor cocktail. The suspension was centrifuged at 15,000 rpm for 20 min at 4 °C. The protein contents were determined using a BCA protein assay kit (Pierce™ BCA Protein Assay Kit, Thermo Scientific). An equal amount of protein from each sample was separated on Criterion™ Precast Gel Tris-HCl (Biorad, Hercules, CA, USA) and transferred to polyvinylidene fluoride membranes (Millipore Corporation). The membranes were blocked for 2 h with a solution containing 5% fat-free milk PBS with 0.1% Tween 20 (Sigma-Aldrich) at room temperature, and incubated overnight at 4°C with each of the following antibodies: anti-p53 antibody (1:1000 Santa Cruz, Dallas, TX, USA), anti-Bax (1:1000 Cell Signaling, Danvers, MA, USA), anti-Casp-3 (1:1000 Cell Signaling), anti-Casp-9 (1:1000 Cell Signaling), anti-Rho (1:1000 Millipore Corporation), anti-Vinculin (1:1000 Thermo Scientific). After washing, the membranes were incubated for 1 h at room temperature with horseradish-peroxidase-conjugated secondary antibody. Densitometric analysis of proteins on western blot were performed with Quantity One software version 4.6.9 by Bio-Rad Laboratories.

### 4.4. Drug Testing

For drug assessment 10,000 cells were seeded per well in 96-well plates. Cells were allowed to recover for three days and then treated. Doses of ifosfamide, epirubicin and eribulin were selected on the basis of plasma levels from pharmacokinetic clinical data. As ifosfamide is used at high doses for the treatment of STS patients, it was administered to the primary culture at the concentration of 100 µM [25,26]. Epirubicin 2 µg/mL, clinical studies have indicated that the C_max_ of epirubicin is between 2 and 3.7 µg/mL [27,28,29]; epirubicin 2 µg/mL plus ifosfamide 100 µM; eribulin 371 ng/mL which corresponds to the plasma peak concentration in patients with solid tumors [30]. Percentages of survival were assessed by MMT assay (Sigma-Aldrich) after 72 h of drugs exposure, following the manufacturer’s instructions as previously described [31]. Two independent experiments were performed. 

### 4.5. Tunel Assay

DNA fragmentation resulting from apoptotic signals was detected by the terminal deoxynucleotidyl transferase (TdT) tunel assay following the manufacturer’s instructions with slight changes [32].

After the treatments, cells were washed twice with PBS, fixed in 1% formaldehyde on ice for 15 min, and incubated in 70% ice cold ethanol for 1 h.

The cells were washed twice in PBS, permeabilized with 0.1% Triton-X100 for 5 min on ice and incubated TdT and FITC conjugated dUTP deoxynucleotides 1:1 solution (Roche Diagnostic GmbH, Mannheim, Germany) in a humidified atmosphere for 90 min at 37 °C in dark conditions.

Images were captured with an inverted fluorescence microscope.

### 4.6. Cell Migration Assay

Scratch wound assay was used for evaluating LPS primary culture cells migration ability after treatment. Briefly, 2 × 10^5^ cells were cultured in a 24-well plate, and exposed to eribulin. Immediately after the treatment, a uniform cell-free area was created by scratching a confluent monolayer with a scraper. The cell migration rate was determined by observing the wound closure after 72 h of treatment and compared with control samples [33].

### 4.7. Statistical Analysis

Three independent replicates were performed for each experiment. Data are presented as mean ± SD, or mean ± SE, as reported, with *n* indicating the number of replicates. Differences between groups were assessed by a two-tailed Student’s *t*-test, and accepted as significant at *p* < 0.05.

## 5. Conclusions

In conclusion, our studies demonstrated that eribulin exerts an antiproliferative and cytotoxic activity through the arrest of cell motility and the induction of apoptosis in ALT/DDLPS. The apoptotic mechanism was found to be mediated by the activation of the p-53 and Bax cascade. Apoptosis may also be related to the activation of Casp-3 and Casp-9, while Rho downregulation indicated the suppression of cell migration. On the basis of these findings, eribulin represents an effective anticancer agent for the treatment of ALT/DDLPS.

## Abbreviations

The following abbreviations are used in this manuscript:

ALT/DDLPS	atypical lipomatous tumor/dedifferentiated liposarcoma
Bax	apoptosis regulator Bax
Casp-3	caspase-3
Casp-9	caspase-9
CDK4	cyclin-dependent kinase 4
CHMP	Committee for Medicinal Products for Human Use
CT	Computed tomography
DDIT3	DNA damage inducible transcript 3
DDLPS	dedifferentiated liposarcoma
EMA	European Medicines Agency
EORTC	European Organisation for Research and Treatment of Cancer
FDA	Food and Drug Administration
FISH	fluorescence in situ hybridization
GLI	glioma-associated oncogene homolog 1 (zinc finger protein)
HE	haematoxylin and eosin
HMGIC	high-mobility-group protein gene
LPS	liposarcoma
MDM2	mouse double minute 2 homolog
MLPS	myxoid liposarcoma
PLS	pleomorphic liposarcoma
Rho	rho family of GTPases
STS	soft tissue sarcoma
WHO	World Health Organization
